# Implementation of a Collaborative HIV and Hepatitis C Screening Program in Appalachian Urgent Care Settings

**DOI:** 10.5811/westjem.2018.9.39512

**Published:** 2018-10-09

**Authors:** Carmen N. Burrell, Melinda J. Sharon, Stephen M. Davis, Elena M. Wojcik, Ian B.K. Martin

**Affiliations:** West Virginia University School of Medicine, Department of Emergency Medicine, Morgantown, West Virginia

## Abstract

**Introduction:**

With the current hepatitis C (HCV) epidemic in the Appalachian region and the risk of human immunodeficiency virus (HIV) co-infection, there is a need for increased secondary prevention efforts. The purpose of this study was to implement routine HIV and HCV screenings in the urgent care setting through the use of an electronic medical record (EMR) to increase a provider’s likelihood of testing eligible patients.

**Methods:**

From June 2017 through May 2018, EMR-based HIV and HCV screenings were implemented in three emergency department-affiliated urgent care settings: a local urgent care walk-in clinic; a university-based student health services center; and an urgent care setting located within a multi-specialty clinic. EMR best practice alerts (BPA) were developed based on Centers for Disease Control and Prevention (CDC) guidelines and populated on registered patients who qualified to receive HIV and/or HCV testing. Patients were excluded from the study if they chose to opt out from testing or the provider deemed it clinically inappropriate. Upon notification of a positive HIV and/or HCV test result through the EMR, patient navigators (PNs) were responsible for linking patients to their first medical appointment.

**Results:**

From June 2017 through May 2018, 48,531 patients presented to the three urgent care clinics. Out of 27,230 eligible patients, 1,972 patients (7.2%) agreed to be screened for HIV; for HCV, out of 6,509 eligible patients, 1,895 (29.1%) agreed to be screened. Thirty-one patients (1.6%) screened antibody-positive for HCV, with three being ribonucleic acid confirmed positives. No patients in either setting were confirmed positive for HIV; however, two initially screened HIV-positive. PNs were able to link 17 HCV antibody-positive patients (55%) to their first appointment, with the remainder having a scheduled future appointment.

**Conclusion:**

Introducing an EMR-based screening program is an effective method to identify and screen eligible patients for HIV and HCV in Appalachian urgent care settings where universal screenings are not routinely implemented.

## INTRODUCTION

Hepatitis C virus (HCV) significantly increases the risk of developing hepatocellular carcinoma and liver cirrhosis.^1^ Treatment of HCV-related illnesses is estimated to cost approximately $6.5 billion per year in the United States (U.S.).^2^ Individuals born between 1945 and 1965 currently account for three-fourths of all HCV infections and are recommended to have at least one HCV test in their lifetime, according to Centers for Disease Control and Prevention (CDC) recommendations.^3^ Recently, an HCV epidemic related to injection of opioids has led to a sharp increase in incident cases in the U.S.

Central Appalachia (Kentucky, Tennessee, Virginia, West Virginia) has been particularly hard hit by this epidemic, with observed cases of HCV increasing 364% between 2006 and 2012.^4^,^5^ A recent study has demonstrated a need for further HCV testing and intervention in the Appalachian region.^6^ Among Central Appalachia states, West Virginia currently has the second-highest incident rate of HCV in the nation.^4^ Of particular concern is the fact that HCV co-infection has been observed in rates as high as 90% among human immunodeficiency virus (HIV) positive injection drug users.^7^ Although West Virginia has historically had a low HIV prevalence, there has recently been an alarming increase of HIV cases in the state.^8^ This increase, coupled with the HCV epidemic, demonstrates the need for established screening efforts to help halt the cycle of transmission of HIV and HCV.^7^

**Population Health Research Capsule**

What do we already know about this issue?

*There is a need for increased Hepatitis C (HCV) testing in conjunction with CDC guidelines in the Appalachian region, due to the high co-infection rates among HIV-positive injection drug users*.

What was the research question?

*Is an electronic medical record-based screening program an effective method to identify and screen eligible patients for HIV and HCV in urgent care centers*?

What was the major finding of the study?

*HIV and HCV screenings are feasible during routine urgent care patient visits, with subsequent successful linkage to care efforts for positive patients*.

How does this improve population health?

*Early identification and intervention of HCV infections may decrease the spread of the virus while in early stages, reduce HIV co-infection rates, and prevent future epidemics*.

One plausible location to increase our HIV and HCV screenings are local, acute care, walk-in clinics where research has been limited. A recent review of the National Hospital Ambulatory Medical Care Survey revealed that HIV testing was significantly greater in outpatient ambulatory medical care settings than in emergency departments (ED) and physicians’ offices, suggesting that urgent cares may be an important setting in which to expand testing.^9^–^10^ HCV has been identified in individuals outside of current CDC recommendations for testing, indicating a need to implement universal screening during patient visits.^11^ Multiple studies have demonstrated success in using a best practice alert (BPA) model, prompting and streamlining the linkage-to-care process.^11^,^12^ Additionally, urgent care clinics may be an ideal setting for both HIV and HCV screenings, as physicians may be less constrained by time or patient acuity compared to the ED setting. To our knowledge, there are no prior scholarly works discussing the implementation of a dual HIV-HCV screening program within an urgent care location, especially within rural Appalachian settings.

The purpose of our study was to implement an electronic medical record (EMR)-based HIV and HCV screening program at three of our local urgent care clinics with the primary objective of using BPAs to enhance a provider’s likelihood of ordering a test in patients eligible for HIV and HCV screenings. A secondary objective was to increase the overall number of tests ordered, adapting from very minimal to routine testing practices.

## MATERIALS and METHODS

### Study Population and Clinical Sites

The three locations used for the implementation of HIV and HCV screenings were two local urgent care clinics (one stand alone and one multi-specialty based) and a student health services clinic affiliated with a large, mid-Atlantic university. The urgent care clinic typically sees approximately 24,000 patients per year of all ages, with an average throughput time of 49.7 minutes. The student health services clinic evaluates approximately 70% of the total student population (~30,000 per year), with an average throughput time of 36.8 minutes. Student health services also has approximately 2,000 visits from the general public per year, including university faculty and staff. Approximately 2–7% of patients seen in these clinics will have blood drawn as a part of their care, although a larger proportion receives point-of-care testing. None of the three walk-in clinics had previously conducted preventive screenings during routine patient visits.

The three sites represent different demographics. Although the majority of patients at all three locations have private-payer insurance (roughly 50%), the percentage of Medicare/Medicaid vs. self-pay varies between the three locations and may have affected screening rates. However, all screenings were free of charge to patients at all three locations, regardless of their insurance status. HIV and/or HCV screenings were only performed during a patient visit if concerns were identified related to current symptoms or when a patient presented for a sexually transmitted infection screening. Therefore, the introduction and implementation of HIV and HCV screenings into the urgent care settings would allow for these tests to become a routine part of patient visits, no longer relying on the clinician-driven method previously used. This study was given a non-human subjects research designation by our university’s institutional review board.

### The Electronic Medical Record

To introduce routine HIV and HCV screenings into the urgent care settings, the EMR (Epic® 2015, Epic Systems Corporation) was used. BPAs, a clinical decision tool, were used to populate within the charts of registered patients who qualified to receive the following: 1) only an HIV screening; 2) only an HCV screening; or 3) both an HIV and HCV screening. The BPAs were developed based on CDC screening guidelines, which include a variety of risk factors.^13^,^14^ HIV testing is recommended for patients aged 13–64 years at least once a year as part of routine healthcare.^14^ A list of recommended guidelines with risk factors warranting HCV screening is shown in the Table.

**Figure 1:**
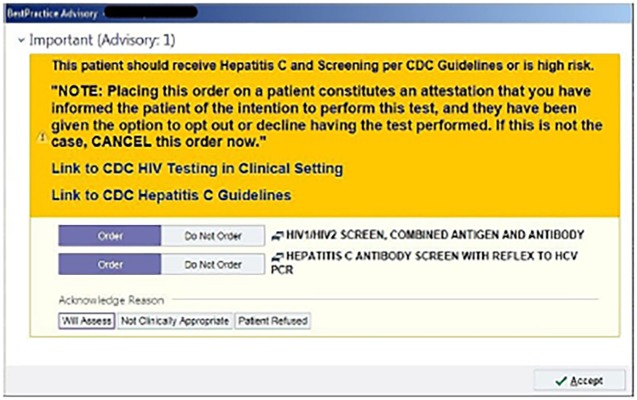
Example of the dual HIV-HCV “best practice alert” that populates upon patient eligibility, which are seen by providers and staff at the urgent care locations. *HIV*, human immunodeficiency virus; *HCV*, hepatitis C virus; *CDC*, Centers for Disease Control and Prevention

The EMR would identify eligible patients by searching charts of registered patients to see if they met screening guidelines and/or had a history of risk factors in the “Problem List” tab. BPAs appeared on the computer screen within the EMR upon opening of eligible patients’ charts during their visit. Upon presentation of a BPA, providers and staff (i.e., physicians, nurses, and technicians) could order the suggested screening tests; if they decided to not order the test(s) for eligible patients, providers and staff were prompted to choose one of the following options: “will assess,” “not clinically appropriate,” or “patient refused” (Figure 1). To prepare for the implementation providers and staff received education on both the BPAs and the screening eligibility criteria at staff meetings .

### Implementation of Screenings

Routine EMR-based HIV and HCV screenings began in June 2017 and were free of charge to all eligible patients. Placards were hung in care rooms, triage areas, and restrooms to inform patients of the current screenings, providing them the opportunity to opt out from testing. If eligible, providers and nursing staff discussed the options of screenings privately with patients in their respective treatment rooms. In addition to having the option to opt out from HIV and/or HCV testing, other exclusions included providers’ decisions on the populated BPAs, and patients refusing a venipuncture during their visit. If patients refused a blood draw, an option for a third-generation oral fluid HIV antibody test at the student health services clinic was offered, with results available within 20 minutes. Patients also had the option to opt out from the oral fluid antibody test, if desired.

Upon patient verbal consent, blood samples or oral swabs were obtained from eligible patients for HIV and/or HCV testing. The HIV screening test used is a fourth-generation combined antigen and antibody chemiluminescent immunoassay test that reflexes automatically to an antibody differentiation immunoassay. All positives obtained from the rapid testing are confirmed with the combined antigen and antibody testing by the laboratory. The HCV screening test used is a chemiluminescent microparticle immunoassay performed on ARCHITECTi®. The test reflexes automatically to quantitative HCV ribonucleic acid (RNA) testing if the initial test result is positive. Results were available within 12 hours.

### Patient Navigators and Linkage to Care

Patients were initially contacted by the urgent care provider with results and follow-up instructions. Upon receiving notification of a positive HIV and/or HCV screening result in the EMR “in-basket” pool, PNs then were responsible for linking patients to their appropriate care needs. PNs would call patients via phone to discuss 1) that patients had spoken to a provider and were aware of their results; 2) the availability of follow-up appointment options; and 3) scheduling their follow-up appointment with the appropriate clinic.

#### HCV Linkage-to-care Process

Patients were referred for follow-up appointments with a university-based, infectious diseases clinic upon an initial HCV antibody-positive screening result. Regardless of confirmatory testing status, it can be helpful to counsel patients about risk-factor modification in the event they are currently “negative” for HCV infection. Therefore, PNs would make initial contact with HCV antibody-positive patients regardless of confirmatory-test outcomes. Patients could also be referred to a university-based, behavioral medicine and psychiatry clinic or a digestive diseases clinic, depending on patient preferences or the specified care plan of referring providers.

#### HIV Linkag- to-care Process

Patients who initially screened positive for HIV with a negative or indeterminate confirmatory test were contacted and encouraged to have repeat testing in six weeks due to the risk of early infection. When possible, these patients were scheduled to return to one of our primary locations. PNs were responsible for linking confirmed HIV-positive patients to a university-based, infectious diseases “Positive Health Clinic” for follow-up appointments.^15^ The Positive Health Clinic provides comprehensive, primary HIV care services to a largely rural, impoverished, medically underserved area, where access to care is limited.

#### Transportation Assistance

In order to support patient linkage to care, PNs offered transportation assistance and coordination with follow-up clinic schedulers. PNs would coordinate taxis with patients who did not have their own means of transportation or provide information on local bus transit routes close to their residence. In addition, PNs would offer gasoline gift cards to those who had a reliable source of transportation but needed transportation assistance. In terms of scheduling, PNs worked closely with clinic schedulers in university-based departments of infectious diseases, and behavioral medicine and psychiatry, and in the urgent cares to quickly get patients into follow-up appointments.

### Data Analysis

We analyzed collected data descriptively to assess the progress of the implementation with the goal to provide feedback to provider and nursing staff. Reports on BPA firings were also conducted via the EMR to provide feedback to staff. We tracked counts of the number of patients tested at each site, as well as counts of the number of positive test results for HIV and HCV. Rates of positivity for HIV and HCV screenings were calculated, as well as the linkage-to-care rates for all positive patients identified at all locations.

## RESULTS

Prior to implementation, approximately 1,639 HIV screenings and 150 HCV screenings were conducted at the clinics between June 2016—May 2017. The majority of HIV screenings were rapid tests (86%) and occurred at the student health services clinic (89%). From June 5, 2017—May 31, 2018, a total of 48,531 patients presented to the three urgent care clinics, with the majority (51%) presenting to the local, stand-alone urgent care clinic. The multi-specialty urgent care clinic began conducting screenings in February 2018, once it opened in September 2017. The BPAs populated on 36,389 patients eligible for HIV screening (75%). Overall, 3,388 patients (9.3%) refused HIV screenings, with 5,771 patients (15.9%) deemed “not clinically appropriate” through the BPAs by providers. Additionally, the BPAs populated on 7,465 patients eligible for HCV screening (24%), with 489 patients (4.2%) deemed “not clinically appropriate’”by providers. Furthermore, a total of 467 patients (4.0%) refused HCV screenings.

**Figure 2:**
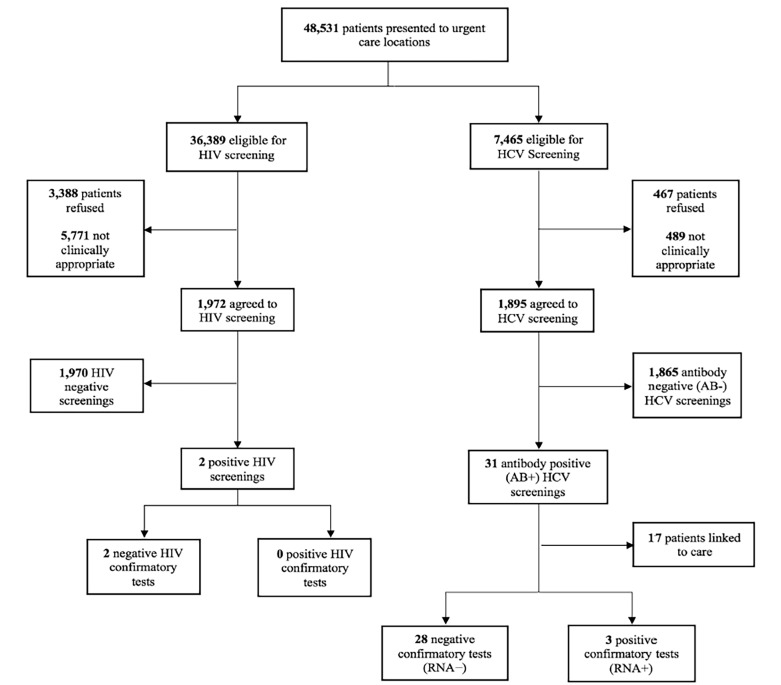
Flowchart of HIV and HCV screenings at all urgent care clinics. *HIV*, human immunodeficiency virus; *HCV*, Hepatitis C virus; *RNA*; ribonucleic acid.

Of the remaining 27,230 patients eligible, 1,972 agreed to be screened for HIV (7.2%). Similarly, of the remaining 6,509 patients eligible, 1,895 (29.1%) agreed to be screened for HCV (Figure 2). The student health services clinic had higher screening rates for both HIV and HCV compared to the local urgent care clinics, with 70% of all screenings occurring at student health. Since screenings were not included as part of the acute sick visit, no initial baseline numbers are available for preventative health screenings in comparison.

Thirty-one patients (1.6%) screened antibody-positive for HCV, with three (9.7%) subsequently having a positive RNA result. The average age of HCV antibody-positive patients was 25 years, ranging from 18–65 years. All patients with antibody-positive HCV results were referred to infectious diseases for follow-up through our PNs. No patients in any of the three clinics were confirmed positive for HIV during this time period. However, two patients had an initial positive screen with a negative confirmatory result. Our PNs were able to link 17 patients (55%) to their first appointments during this time, with the remaining 14 (45%) patients having a scheduled future appointment.

## DISCUSSION

Our study demonstrated that an EMR-based HIV and HCV screening program is effective in the Appalachian urgent care settings. The “opt-out” model of testing allowed these varying locations to successfully increase screenings in conjunction with CDC guidelines and increase linkage to care through the use of PNs. The EMR is effective at identifying eligible patients to be screened for HIV and HCV, as demonstrated in the number of BPA firings during the initial four months.

During the initial implementation, there were relatively low acceptance rates for both HIV and HCV testing by our patient population. There are a number of factors as to why initial patient testing was low. If providers and staff were choosing “will assess” upon firing of the BPAs, instead of immediately addressing it, the BPAs would continue to populate on the same patients until one of the following occurred: the test was ordered; it was considered not clinically necessary by the provider; or the patient refused to be tested. In cases where only “will assess” was chosen, it is possible that a final decision for the screenings may not have been addressed, as it was not required for chart closure. In these cases, documentation for reasoning was frequently unrecorded. For those patients who were documented as “not clinically appropriate” or “patient refusal,” some indicated that they would defer testing, since no additional blood work was indicated at the time of visit.

With a low percentage of patients typically having blood drawn during a visit and short throughput times, patients do not want to spend the “extra” time giving blood. A practical alternative would be to reinforce the availability of the oral swab test to those patients, which could help increase the HIV testing rates. Other patients indicated a desire to discuss testing with their primary care provider or felt they were low risk and testing was unnecessary. Finally, due to the strong, negative stigma still surrounding HIV and HCV, patients may not want to know if they are positive for either. In some cases, the tests were ordered but not directly from the BPA if later decided. Although there was a comment option on the BPA, it was rarely used by the providers to capture the additional reasons noted above. It may be beneficial to require reasoning on the BPA in order to close the patient chart, as well as enforce consistent responses from all providers and nursing staff, to generate the most accurate reports of the BPA results.

BPA fatigue is often a problem in clinical locations due to multiple documentation requirements. In the urgent care settings, these BPAs are somewhat limited when compared to inpatient services and outpatient primary care. Our current EMR administration has addressed some of these needs in the background that are not readily apparent to the practitioner. In these cases, certain documentation is required prior to chart closure without prompting a BPA alert. Due to this, our location was likely more successful than others by implementing this method. Although we did not survey staff perceptions about the implementation of routine HIV and HCV testing, physicians, advanced practice providers, and nursing staff seemed willing to participate when education of the department was performed during departmental meetings. Testing was performed under all providers and orders seemed to increase with ongoing education.

There were a number of challenges with the EMR. First, the accuracy and completeness of searchable, historical data in the EMR affected the accuracy of BPA firings. If the patient’s past medical history or current problem list was not up to date, BPAs would populate unnecessarily or repeatedly in the case of those patients who had been previously tested. Risk factors that were not captured by the EMR, such as multiple sexual partners and injection drug use, represented missed opportunities for screening. These behaviors were often not addressed in the patient visits unless indicated by initial patient complaints. If addressed at a previous visit, the information may have been documented within the body of the provider note as free-text and not in a location that could be easily accessed via the EMR logic for the BPA requirements. Also, BPAs were initially set to only detect prior blood screenings. During this study period, past oral HIV antibody testing was not captured by the BPAs. However, upon review of the BPA data, patients who had refused initial blood work but consented for an oral HIV antibody swab were not counted in the totals; therefore, in future we would like to adjust the BPA to capture these tests.

Challenges with EMR data have been previously reported in the literature.^16^,^17^ Despite the commonly held belief that EMRs decrease medical errors by providing complete patient information and history, inaccuracies and incompleteness are a frequently occurring problem. One study found that 25% of patient charts were incomplete, with the most commonly inaccurate fields being current medications, medical history, and medical allergies.^16^ In a study by Tse and You, inaccuracies in medications were reported in 51% of records.^17^ While over 91% of participants had a history summary with eight or less items present, omissions were reported for one in every five participants.^17^ Further work is needed to improve EMR accuracy, especially when implementing widespread, EMR-based routine screening for infectious diseases.

Since the BPAs were designed around CDC guidelines for screenings, there is the possibility that some patient populations could be missed. Of particular interest are those who are younger than the HCV birth cohort of 52–72 years of age. Although baby boomers account for the majority of existing infections, newly diagnosed HCV infections are increasing most rapidly among 20–29 year olds.^14^,^18^ The urgent care providers were recognizing risk factors not previously noted in the EMR, and thus began using wider screening efforts than the original BPA design. For example, if the BPA was triggered by patient eligibility for an HIV screening, providers would also recommend an HCV screening to the patient, since they would already be having blood drawn upon verbal consent. Interestingly, all of the HCV antibody-positive patients were younger than the birth cohort, with an average age of 24 years, ranging from 18–38 years. This may be related to the HCV epidemic that the Appalachian region is currently enduring. The initial findings support the need for universal HCV screenings among this population, since birth cohort screening does not identify a significant portion of people infected with HCV.^18^

PNs played a crucial role in the screening program. All 10 patients (100%) who tested HCV-antibody positive were successfully scheduled with referrals to infectious diseases for follow-up. Although 90% of those patients did not have HCV RNA-positive results, it is important for the patients to attend their follow-up appointments due to possibly having a previous infection. These follow-up appointments also present an opportunity to counsel “negative” patients on risk-factor modification. Our linkage-to-care rate was significantly higher than what is currently seen in the literature.^19^,^20^ In a recent EMR review from a large healthcare system, no action was taken in 30% of patients who tested positive for HCV.^16^ Other studies have demonstrated that only about 15% of patients diagnosed with chronic HCV have received treatment.^20^ A possible explanation for our successful linkage-to-care rate could be the PN transportation assistance. Transportation is a significant issue with patients in the Appalachian population; therefore, providing financial assistance has benefitted our population tremendously.

Their successes notwithstanding, the PNs have faced a number of challenges. PNs discovered that patients were more likely to attend their infectious diseases follow-up appointments if scheduled close to their original urgent care visit. Initially, PNs and schedulers could get patients in within four weeks of initial visit. However, both PNs and schedulers have become more efficient in scheduling these appointments closer to within two weeks of initial visit. This has been a common issue for patient care coordinators in other settings; the longer the delay to getting a follow-up appointment, the less likely a patient is to attend.^18^ Patients who are motivated to seek treatment will sustain this wait; however, those patients who lack motivation or education are less likely to wait and will eventually fail to access care.^18^ Additionally, PNs could only interface with patients when contact information was available to them in the EMR. Therefore, if patients did not provide sufficient or correct contact information upon initial presentation to the clinic, the PNs could not follow up with them in a timely manner, if at all. To improve follow-up we suggest that clinic staff encourage patients to provide multiple modes of contact upon registration with front-desk staff at these clinics.

### Future Directions and Improvements

Although initial implementation has been successful, there are many areas to improve and expand upon. First, patient-reported reasons for not having HIV and/or HCV screenings conducted during their visit should be documented in provider and staff notes within the patients’ EMR. This would allow tracking of patient perceptions. Similarly, surveying all patients on their opinions of HIV and HCV screenings during their visits, regardless of whether or not they were tested, would increase insight into patient perceptions. This could provide feedback on the opt-out process for testing, as well as on the placards hanging in all treatment rooms and triage locations. Surveying providers and staff on their opinions and perceptions of the screening program would be valuable for improving the screening process. It is crucial to continuously gain feedback from those on the front lines of implementation in order to best tweak the program to what will be most efficient for both the patients, and the providers and nursing staff. It is also important to have multiple risk factors recorded in easily accessible areas of the EMR so the BPA will populate accurately.

## CONCLUSION

This study has demonstrated the feasibility of introducing an EMR-based method to identify and screen eligible patients for HIV and HCV in Appalachian urgent care settings, successfully transitioning from conducting essentially no screenings to making this a part of routine patient visits within a 12-month period. Other urgent or acute care clinics in the Appalachian region should consider adopting a similar practice to manage the side effects of the current opioid epidemic.

## Figures and Tables

**Table:** Hepatits C screening recommendations and risk factors, per CDC guidelines.^13^

Guidelines	Risk factors
HCV testing is recommended for those who:	Are adults born from 1945 through 1965 (without prior ascertainment of HCV risk factors) Are currently injecting drugs Ever injected drugs, including those who injected once or a few times many years ago Have certain medical conditions, including persons: who received clotting factor concentrates produced before 1987 who were ever on long-term hemodialysis with persistently abnormal alanine aminotransferase levels (ALT) who have HIV infection Were prior recipients of transfusions or organ transplants, including persons who: were notified that they received blood from a donor who later tested positive for HCV infection received a transfusion of blood, blood components, or an organ transplant before July 1992
HCV testing based on a *recognized exposure* is recommended for:	Healthcare, emergency medical, and public safety workers after needle sticks, sharps, or mucosal exposures to HCV-positive blood Children born to HCV-positive women

*HCV*, Hepatitis C virus; *CDC*, Centers for Disease Control and Prevention.
